# Effect of Proteins on the Vulcanized Natural Rubber Crosslinking Network Structure and Mechanical Properties

**DOI:** 10.3390/polym16212957

**Published:** 2024-10-22

**Authors:** Yueqiong Wang, Shiqi Su, Hongchao Liu, Rui Wang, Lusheng Liao, Zheng Peng, Jihua Li, Haijun Wu, Dongning He

**Affiliations:** 1 Guangdong Provincial Key Laboratory of Natural Rubber Processing, Agricultural Products Processing Research Institute, Chinese Academy of Tropical Agricultural Sciences, Zhanjiang 524001, China; 2 Key Laboratory of Advanced Materials of Tropical Island Resources, Ministry of Education, School of Materials Science and Engineering, Hainan University, Haikou 570228, China; 3 School of Chemistry and Chemical Engineering, Lingnan Normal University, Zhanjiang 524048, China

**Keywords:** natural rubber, proteins, crosslinked network structure, mechanical properties

## Abstract

Proteins are important factors affecting the properties of natural rubber. Therefore, investigating the effect of free and bonded proteins on the structure and mechanical properties of the vulcanized crosslinking network of natural rubber would provide a theoretical basis for the production of high mechanical resistance natural rubber. Herein, natural rubbers with different protein contents and types were prepared by high-speed centrifugation. And, the effects on their network structure, vulcanization, tensile strength, tearing strength and dynamic mechanical properties were investigated. The results showed that the reduction in protein content led to the decrement in the entanglement networks, crosslinking density and tensile and tear strengths of the vulcanized natural rubber. Moreover, the bonded proteins had an obvious influence on the vulcanization process, while free proteins played an important role in the crosslink densities. These results reveal that both bonded and free proteins are involved in the vulcanization process and the construction of the vulcanized crosslinking network structure of natural rubber, which enhances the mechanical properties such as the modulus and tensile strength of vulcanized natural rubber.

## 1. Introduction

Natural rubber (NR), an important engineering material, is a natural polymer synthesized from fresh latex collected from Brazilian three-leaf rubber trees. NR occupies an indispensable position in aerospace, rail transportation and medical and industrial fields for its excellent physical and mechanical properties [1,2]. The outstanding properties of NR are due to the non-rubber components that contain proteins and lipids. Both the proteins and lipids can affect the terminal molecular chains of NR. It is widely known that the non-rubber components have a significant effect on the network structure of NR. Tarachiwin et al. [3,4] demonstrated that proteins coupled to the ω-terminal group of the rubber chains, linked by hydrogen bonds, and phospholipids coupled to the α-terminal group either by hydrogen or ionic bonds. The aggregation of proteins and phospholipids then gradually formed a crosslinked network of natural rubber. Dixit et al. [5] showed that proteins in rubber were attached to each other in the form of hydrogen bonds to form crosslinking points to the ω-terminal end of the rubber molecular chain, while phospholipids formed branched structures at the α-terminal end of the rubber molecular chain. Former research proved that both proteins and lipids formed a “naturally occurring network” in unvulcanized NR [6,7].

In the non-rubber components, protein content is about 2.2% in NR latex [8], which is an important part. Research of proteins’ effect on the network and mechanical properties of NR has been widely reported [6,9,10]. It has been found that the mechanical properties of NR dramatically decrease for the removal of proteins. Generally speaking, the proteins in NR are usually removed by centrifugation, proteases or urea. There are two main forms, called binding proteins and free proteins, in NR latex. The bonded proteins interact with the rubber particles and are distributed on the surface of the rubber particles, while the free proteins are mainly found in the serum of the natural latex. It is known that proteins affect the structure of the vulcanized crosslinking network of NR [11,12,13], which influences the properties of vulcanized NR [14,15,16]. Huang et al. [17] reported that protein content could be considered as the guidance for the selection of NR and pointed out the importance of proteins. There is also research focusing on the effect of exogenous proteins on the mechanical properties of NR. It has been found that adding exogenous proteins improves the mechanical properties and decreases the heat generation [18,19,20].

However, previous studies have illustrated the important influence of proteins on the structure and mechanical properties and network of NR. The contributions of the two types of endogenous proteins in NR are rarely discussed. As is known, the two types of proteins in NR are due to their types existing in NR. Proteins dispersed in NR latex that could be easily removed by a physical method are called free proteins. Those that are linked to the molecular chains of NR by functional groups are called bonded proteins. However, there is little research about the effects of bonded and free proteins on the structure and properties of NR. But, there are definitely different effects of the two types of proteins on the mechanical properties from our previous studies [21]. Free and bonded proteins and their contents have different influences on the structure and properties of the vulcanized crosslinking network of NR, but their influences have not been specially discussed. As is known, network structure is a crucial factor influencing NR’s mechanical properties. Therefore, both the network structure and mechanical properties affected by the two types of proteins are necessary to study.

In this study, NR latex with different contents and existing types of proteins were prepared by high-speed centrifugation. Moreover, vulcanized NR was prepared by a mechanical mixing method, and the effects of the free and binding proteins on the crosslinking network and mechanical properties of the natural rubber were analyzed. The results will give advice on tuning the network structure and mechanical properties of NR.

## 2. Materials and Methods

### 2.1. Sample Preparation

#### 2.1.1. Preparation of Natural Rubber Latex (NRL)

**Figure 1:**
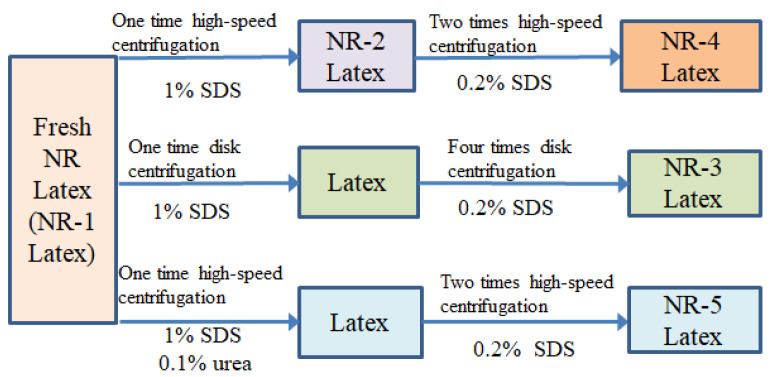
Preparation process of NRL with different protein contents.

Fresh NRL (dry rubber content 35%) was provided by Guangdong Guangken Maoming Rubber Group Co., Ltd., Maoming, China. The NRL was added with 0.6% (*w*/*w*) ammonia at room temperature. Urea solution at 10% concentration and sodium dodecyl sulfate (SDS) solution at 10% concentration were prepared to be used with fresh latex. NRL with different protein contents were prepared using disc centrifugation or high-speed centrifugation, and the preparation process is shown in Figure 1. The samples were prepared as follows.

NR-1. The NR-1 was the fresh NRL without centrifugation.

NR-2. For the NR-2, 1% (*w*/*w*) SDS was added to the fresh latex and diluted to a 30% dry rubber content with deionized water, and then the mixture was stirred for 2 h to mix it together. A high-speed freezing centrifuge (CR22N/21N, Hitachi, Tokyo, Japan) was used to centrifuge the paste at 8000 r/min for 30 min at 25 °C, and then the upper cream was collected and diluted to a 60% dry rubber content by adding deionized water and 0.6% (*w*/*w*) ammonia. NRL with high-speed centrifugation conducted once removed most of the free proteins in the natural latex and preserved the bonded proteins and some free proteins.

NR-3. Fresh NRL with 1% (*w*/*w*) SDS solution was centrifugated using a disc centrifuge (JRLDR200, Yixing Haide Separation Machinery Co., Ltd., Yixing, China) 5 times. Every cycle, the centrifugation diluted the NRL to a 30% dry rubber content, and 0.2% (*w*/*w*) SDS was added. After the fifth centrifugation, the protein content changed very little. Therefore, the free proteins were completely removed and only the bonded proteins were preserved in the NRL. The N content was 0.099%, which was similar to the previous report [22].

NR-4: The NR-4 was centrifuged one more time than the NR-2. After the first centrifugation of the NRL, the upper cream was diluted to a 30% dry rubber content and 0.2% (*w*/*w*) SDS was added. After the second centrifugation, the upper cream was diluted to a 60% dry rubber content by adding deionized water and 0.6% (*w*/*w*) ammonia.

NR-5: For the NR-5, 0.1% urea and 1% SDS were added to the fresh NRL; then, deionized water was added to dilute it to a 30% dry rubber content, and it was mechanically stirred for 2 h to mix it well. The urea-treated natural latex was centrifugated at 8000 r/min for 30 min at 25 °C using a high-speed refrigerated centrifuge, and the upper cream was diluted with 0.2% SDS and deionized water and centrifuged twice. Since urea-treated natural latex can remove bonded proteins, it can be compared with the samples treated three times by the high-speed centrifugation. The NR-4 can be used to compare the effects of different bonded protein contents on the properties of natural rubber.

#### 2.1.2. Preparation of Vulcanized NR

The NR-1, NR-2, NR-3, NR-4 and NR-5 latex were poured into a glass mold and dried at 40 °C to a constant weight. The formula was as follows.

Zinc oxide (ZnO) at 6 parts per hundred of rubber (phr), sulfur (S) at 3.5 phr, stearic acid (SA) at 0.5 phr and accelerator NS at 0.5 phr were used. An open two roller mill was used to mix the natural rubber and the agents together, and then the uniform NR compounds were obtained.

A moving die rheometer (MDR-2000E, Huzhou Hongqiao Rubber Machinery Co., Ltd., Huzhou, China) was used to measure the t_90_ at 143 °C. After that, the NR compound was vulcanized for (t_90_ + 5) min on a plate vulcanizer to obtain the vulcanized NR.

### 2.2. Sample Analysis

#### 2.2.1. Fourier Transform Infrared (FTIR) Spectroscopy

The NRL were dried on molds with small holes to obtain films with a thickness of 60 to 80 μm, and the molecular structure of the NR was tested using a FTIR spectrometer (Tensor 27, Bruker, Munich, Germany) in the wavelength range of 400 cm^−1^ to 4000 cm^−1^.

#### 2.2.2. Nitrogen Content

A fully automatic Kjeldahl nitrogen analyzer (Kjeltec 8400, Foss, Denmark) was used to determine the nitrogen content of the NR in accordance with the GB/T8088-2008 standard [23].

#### 2.2.3. Mechanical Properties

A tensile machine (AI-7000-SUI, Gotech Co., Dongguan, China) was used to test the tensile properties at a rate of 500 mm/min in accordance with the GB/T528-2009 [24] standards. Five samples were repeated to obtain the strength parameters. The cyclic tensile test was operated at a rate of 200 mm/min.

#### 2.2.4. Crosslinking Density

The equilibrium swelling method was used to determine the crosslinking density of the vulcanized NR. A sample of about 0.5 g was cut and weighed as *m*_0_, the vulcanized rubber sheet was immersed in a 100 mL toluene solvent for 72 h and then the dissolved rubber was weighed and recorded as *m*_1_. Subsequently, it was put in an oven at 80 °C and dried to a constant weight. Finally, the rubber sheet was removed and cooled down to room temperature and weighed again, recorded as *m*_2_. The crosslinking density was calculated using the following formula.

(1) $$v_{r}=\frac{\frac{m_{2}}{\rho }}{\frac{m_{2}}{\rho }+\frac{\left(m_{1}-m_{0}\right)}{\rho_{s}}}=\frac{1}{Q}$$

(2) $$V_{c}=-\frac{1}{V}\frac{ln(1-v_{r})+v_{r}+\chi v_{r}^{2}}{\sqrt[{3}]{v_{r}}-\frac{1}{2}v_{r}}\;$$

In Equations (1) and (2), Q is the swelling degree of the rubber; $v_{r}$ is the volume fraction of the rubber after swelling; $V_{c}$ is the average number of molecular chains per unit volume, i.e., in mol/cm^3^; ρ and $\rho_{s}\;$ are the densities of the rubber and toluene, respectively, which are 0.913 g/cm^3^ and 0.865 g/cm^3^; χ is the interaction parameter of the natural rubber and toluene, which is 0.393; and *V* is the molar volume fraction of the toluene, which is 106.3 cm^3^/mol.

#### 2.2.5. Dynamic Mechanical Analyzer (DMA)

The samples were cut down to long strip samples and tested using a dynamic thermo-mechanical analyzer (DMA Q800, TA, Newcastle, DE, USA). The temperature range was from −90 °C to 80 °C, and the rate was 3 °C/min.

## 3. Results and Discussion

### 3.1. FTIR Analysis

**Figure 2:**
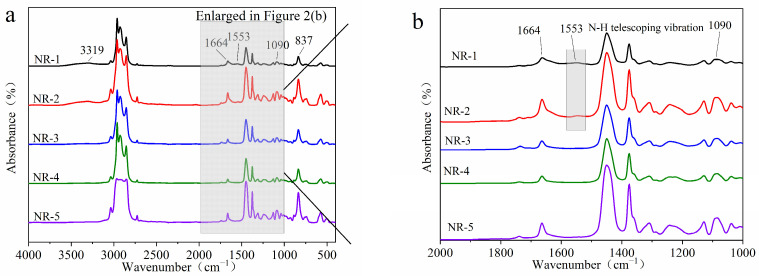
(**a**) Infrared spectrogram and (**b**) Enlarged infrared spectrogramof natural rubber with different protein content.

Figure 2 shows the infrared spectra of the NR gums with different protein contents. It can be seen in Figure 2a,b that the untreated fresh latex, NR-1, and the NR-2 treated one time with high-speed centrifugation have obvious peaks at 3319 cm^−1^ and 1553 cm^−1^, which can be attributed to the N-H telescoping vibration and deformation vibration, respectively, which indicates that there are proteins in NR. As is seen, the 3319 cm^−1^ (Figure 2a) and 1553 cm^−1^ (Figure 2b) peaks in the NR-3, NR-4 and NR-5 almost disappear [21,25]. The peaks of the C=C stretching vibration and the C-H bending vibration are 1664 cm^−1^ and 837 cm^−1^, respectively. As a result, the NR-1 contained all the proteins, which included both free and bonded proteins; the NR-2 lost most of the free proteins after the high-speed centrifugation process; the NR-3 retained all the bonded proteins, and the NR-4 and NR-5 contained only part of the bonded proteins, which also agreed with the reference [22].

### 3.2. Nitrogen Content Analysis

The protein content in natural rubber can be calculated by multiplying the determined nitrogen content by a factor of 6.25 [26]. The nitrogen and protein contents of the NR are shown in Table 1. Free proteins are more easily removed during centrifugation, resulting in a decrease in protein content [6]. The protein content of the NR-2 was reduced from 3.356% to 0.984% after the high-speed centrifugation being conducted once. Disc centrifugation is not as effective as high-speed centrifugation because it is hard to separate the bonded proteins. Therefore, disc centrifugation was used to obtain the NR-3 sample with only bonded proteins, and the nitrogen content of the NR-3 was 0.099%, and the protein content was 0.619%. These data are consistent with the results in the literature [22], which indicates that the NR-3 had all the free proteins removed, and only the bonded proteins were preserved. High-speed centrifugation has a high separation force and can remove both free proteins and bonded proteins. For further removing proteins, additives should be used to depart the bonded proteins [27]. So, urea was used to depart the bonded proteins from the NR because urea can destroy the hydrogen bonding of proteins and increases the solubility of the hydrophobic side chains of proteins in water [28,29]. As a result, the protein content of the NR-5 was significantly decreased to 0.135%.

### 3.3. Effect of Proteins on the Vulcanization Properties of NR

Table 2 shows the vulcanization parameters of the NR with different protein contents. It can be seen that the maximum torque of the NR decreased with the decrement in the protein content. Meanwhile, the scorch time t_10_ and optimum vulcanization time t_90_ increased with the decrement in the protein content as the amide group of the protein can enhance the vulcanization rate and promote the NR forming more crosslinking points that lead to the increment in the maximum torque [30,31]. Based on the existing forms of proteins, it can be seen that the free proteins had a significant effect on the maximum torque M_H_ of the natural rubber, which decreased from 7.14 dN·m to 6.12 dN·m after the removal of free proteins, as the protein content decreased from 3.356% to 0.619%. However, the M_H_ decreased from 6.12 dN·m to 6.04 dN·m after the bonded protein was reduced from 0.619% to 0.135%. In addition, the free proteins showed a significant effect on the t_90_, which was prolonged from 12.2 min to 18.9 min, with a 2.737% (3.356% (the protein content of the NR-1) minus 0.619% (the protein content of the NR-3)) reduction in the free protein. The t_90_ was further prolonged from 18.9 min to 24.1 min after the reduction in the bonded proteins, while the content of the bonded proteins was reduced by 0.063% (0.198% (the protein content of the NR-4) minus 0.135% (the protein content of the NR-5)). Although the t_90_ reduction in the bonded proteins was lower than in the free proteins, the decreasing content of bonded proteins was much smaller than that of the free proteins. As a result, it is indicated that although the bonded protein content was lower in the NR, it had a more significant effect on the t_90_ than the free proteins.

### 3.4. Effect of Proteins on NR Mechanical Properties

**Figure 3:**
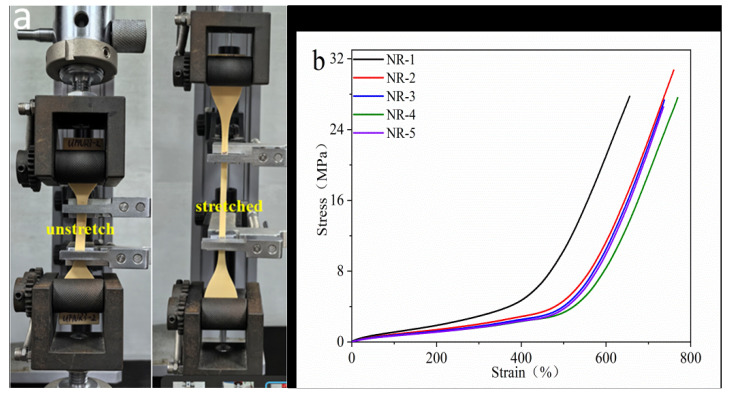
(**a**) Stress–strain process and (**b**) curves of rubber with different protein content.

Table 3 and Figure 3 show the tensile parameters and the stress–strain process and curves of the vulcanized NR. As can be seen in Table 3, the tensile strength of the NR tended to increase and then decrease as the protein content decreased, while the stress decreased. That is because the proteins had obvious influences on the NR crosslinking network. The tensile strength of the NR-2 increased after part of the free protein was removed. As free proteins can form crosslinking networks [32], there is an optimum crosslinking density in vulcanized NR [33]. If the crosslinking density is too large, the molecular weight between the crosslinking points decreases; short chains easily form defects, leading to a decline in tensile strength. After removing some free proteins, the density of the crosslinked network decreases and the number of defects decreases, and then the tensile strength increases. As a result, the tensile strength of the NR-2 was larger than that of the NR-1. With the further removal of the free proteins, the NR-3 contained only bonded proteins, and the crosslinking network formed by the proteins reduced, which led to a decrease in the tensile strength and stress. In addition, the strain-induced crystallization behavior of natural rubber also affects its mechanical properties, which can be reflected by the 500% stress, because vulcanized NR already crystallizes at the strain [34,35]. It is clear that the vulcanized NR exhibited a much higher 500% stress when it possessed more proteins, which was due to the higher crystallinity of the vulcanized NR. Moreover, our previous studies have shown that vulcanized natural rubber has the highest degree of crystallinity and the best tensile properties at a crosslinking density of 1.84 × 10^−4^ mol/cm^3^. In brief, the effect of the proteins on the structure of the crosslinked network is in agreement with our former results [12].

### 3.5. Effect of Proteins on NR Crosslinking Network

Crosslinking density is an important type of data reflecting the structure of vulcanized networks [36]. Table 4 shows the crosslinking density *V_c_* and average molecular weight between the crosslinking points *M_c_* of the vulcanized NR. As can be seen in Table 4, the crosslinking density of the NR decreased with decrement in the protein content, while the average molecular weight between the crosslinking points increased. This is because the proteins in NR can participate in the construction of the crosslinking network by forming crosslinking points, which leads to an increase in the crosslinking density of the networks [32]. It is known that proteins exist in peptide bonds [37]; the carboxylic acid groups and amino groups in peptide bonds can react with zinc oxide to form coordination complexes, which improves the solubility of the zinc oxide in NR. Moreover, coordination complexes enhance the solubility of zinc salts in NR matrices and reduce the activation energy for forming crosslinking. Moreover, the unstable Zn-S bond of the coordination complexes effected by the amino or carboxyl groups can initiate a ring opening of the S_8_ ring, which can act with the molecular chains of NR to form a crosslinking precursor [30]. Then, the crosslinking precursor and NR molecular chains react to form new crosslinking bonds, which promotes the NR vulcanization crosslinking reaction, so the crosslinking density of the NR increases. Therefore, as the protein content decreases, the carboxylic acid groups and amino groups contained in the NR decrease, and the coordination complexes formed by the proteins and zinc salts decrease, slowing down the vulcanization crosslinking reaction of the NR. And, the crosslinking structure of the NR reduces, leading to the crosslinking density decreasing. As can be seen from Table 4, the crosslinking density of the vulcanized NR decreased from 2.35 × 10^−4^ mol/cm^3^ to 1.52 × 10^−4^ mol/cm^3^ after the removal of the free proteins. The crosslinking density decreased from 1.52 × 10^−4^ mol/cm^3^ to 1.46 × 10^−4^ mol/cm^3^ after the bonded protein content was further reduced from 0.619% to 0.135%. The crosslinking density influenced by the bonded proteins was much lower than that influenced by the free proteins. This may be due to the fact that bonded proteins are involved in the construction of physical networks and are less likely to form ligand complexes with zinc salts during vulcanization crosslinking; thus, fewer crosslinking bonds are formed by the bonded proteins, which in turn has less effect on the crosslinking density.

**Figure 4:**
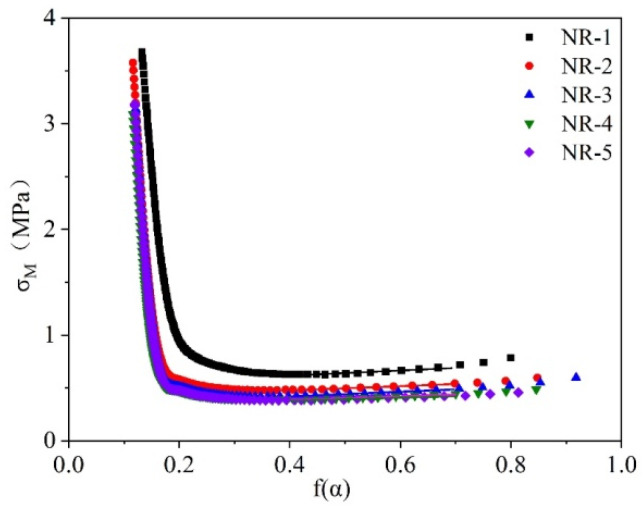
f(*α*)-*σ_m_* diagram of each sample.

The tube model theory was used to calculate the degree of contribution of the topological entanglement and chemical crosslinking network of the vulcanized NR. The effect of the proteins on the structure of the crosslinked network was analyzed using Equations (3) and (4) [38]. The diagrams of each sample based on these equations are shown in Figure 4.

(3) $$\sigma_{M}=\frac{\sigma }{\left(\alpha -\alpha^{-2}\right)}=G_{c}+G_{e}f\left(\alpha \right)$$

(4) $$f\left(\alpha \right)=\frac{\beta }{2}\frac{\alpha^{\beta /2}-\alpha^{-\beta }}{\alpha^{2}-\alpha^{-1}}$$

In Equations (3) and (4), *σ_M_* is the reduced stress; *G_c_* is the elastic modulus created by the chemical crosslinking network (i.e., the effective crosslinking); *G_e_* is the elastic modulus created by the entanglement network; *σ* is the stress; *α* is the elongation ratio of the NR in tension; and *β* is the empirical parameter between the deformed pipeline in tension and the unstretched pipeline at the equilibrium, which is usually taken as 1 [39].

Figure 4 shows the f(*α*)-*σ_m_* curves for each specimen, and linear fitting is operated in the range of 0.4–0.7 of the *X*-axis. The network structural parameters are shown in Table 5, which are the chemical crosslinking modulus *G_C_*, the entanglement network modulus *G_e_*, the effective crosslinking density *V_c_*, the average chain length between the effective crosslinking points *M_c_*, the molecular mean-square end distance between the crosslinking points *R_c_*, the average number of chain segments between the crosslinking points N, the tube radius *d_0_* and the number of statistical chain segments ne between two neighboring effective entanglement points; the parameters were calculated using the same method as in our former research [36,40]. As can be seen from the table, the chemical crosslinking modulus *G_c_* and physical entanglement modulus *G_e_*, as well as the effective crosslinking density *V_c_*, of the NR show a decreasing trend as the content of protein decreases, while the related parameters of the rubber, such as *M_c_*, *R_c_*, *N*, *d*_0_ and *n_e_*, show an increasing trend. This is due to the fact that as the protein content decreases, the coordinated complexes formed by the protein and zinc salts decrease, and the crosslinking points of NR also reduce, resulting in a loosely arranged vulcanization network structure. Therefore, the decrease in the protein content of NR leads to a decrease in the chemical crosslinking modulus and physical entanglement modulus as well as the effective crosslinking density of vulcanized NR, whereas the related quantities such as the molecular weight between the effective crosslinking points of NR increase as the crosslinking points and entanglement points of NR decrease.

### 3.6. Hysteresis Loss

The hysteresis loss hysteresis loop is the energy loss due to the friction within the NR molecules during the stretch–recovery process [41]. NR hysteresis loops are operated by the stretch–recovery process, and the integral hysteresis loop area is the energy loss of the NR in the stretch–recovery process.

**Figure 5:**
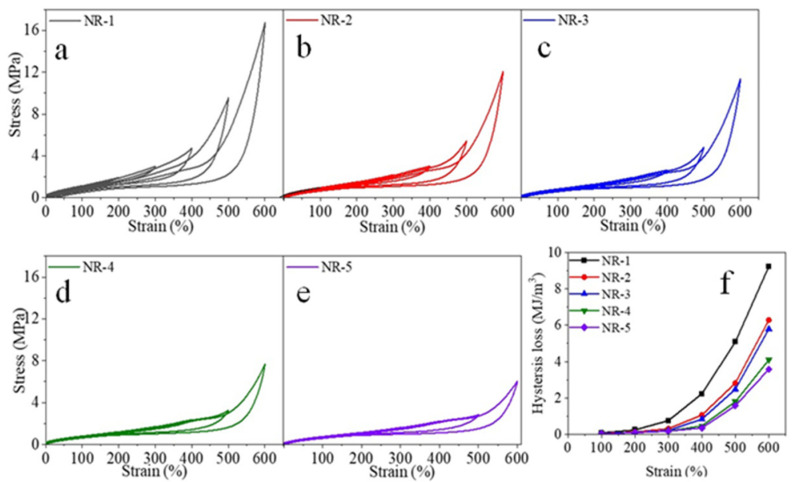
Hysteresis area of (**a**) NR-1, (**b**) NR-2, (**c**) NR-3, (**d**) NR-4, (**e**) NR-5 and (**f**) hysteresis loss of vulcanized NR with different protein content.

Figure 5 shows the tensile recovery curves of the vulcanized NR with different protein contents. From Figure 5a–e, it can be seen that as the strain increased, the hysteresis loop and its area became larger; as the protein content in the NR decreased, the area of the hysteresis loop of the vulcanized NR shrunk and the hysteresis loss decreased at the same strain. This is due to proteins forming network structures with molecular chains in NR, and because the breaking and rebuilding of ionic bonds during stretching causes more energy dissipation [22]. It also can be seen in Figure 5f that after the removal of all the free proteins, the hysteresis loss of the NR-3 vulcanized rubber decreased more significantly than the NR-1 when the strain was more than 300%; after the further removal of the bonded proteins, the hysteresis loss of the NR-4 and NR-5 decreased more obviously than the NR-3 when the strain was more than 400%, and the difference between their hysteresis loss at 300% is not significant. It is indicated that the network structure formed by the free proteins breaks and slips at a 300% strain, whereas the crosslinked network structure formed by the bonded proteins does not break or slip until a 400% strain, resulting a different hysteresis loss.

### 3.7. Effect of Protein on Dynamic and Mechanical Properties of Vulcanized NR

**Figure 6:**
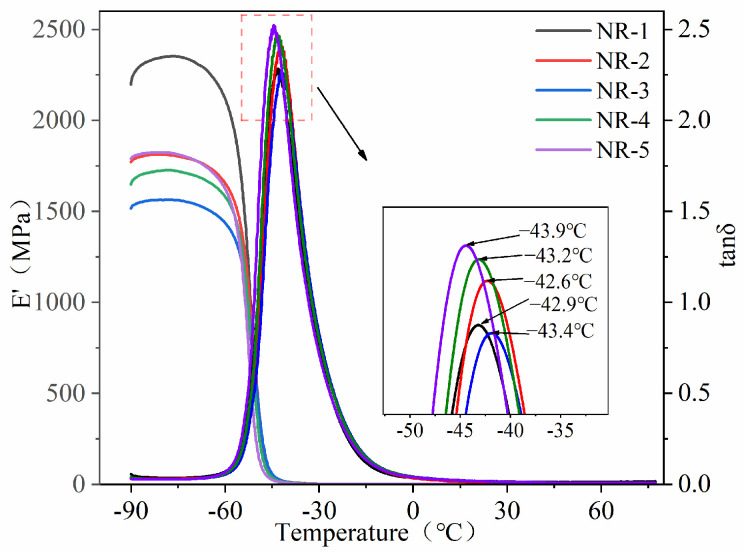
The energy storage modulus of natural vulcanized rubber with different protein content changes with temperature.

Figure 6 shows the energy storage modulus and loss factor versus temperature for the vulcanized NR with different protein contents. As can be seen in Figure 6, the storage modulus E′ of the NR-1 was much higher than that of other vulcanized NR. This suggests that proteins can participate in the crosslinked network construction, which increases the crosslinking density and thus improves the storage modulus and rigidity of vulcanized NR. From the loss factor curves, it can be seen that the *T_g_* of the NR-3 was −43.4 °C, which was slightly lower than that of the NR-1, which was −42.9 °C, whereas after the bonded proteins were further removed, the *T_g_* of the NR-5 was −43.9 °C, which was slightly lower than that of the NR-3. This indicates that both bonded and free proteins are involved in the construction of the crosslinked network, and the removal of these proteins decreases the crosslinking density and increases the mobility of the molecular chains, resulting in a slight decrement in the *T_g_*. Both free and bonded proteins are involved in the construction of the crosslinking network of vulcanized NR.

## 4. Conclusions

In this study, NRL with different forms and contents of proteins were prepared using centrifugation. The effects of the protein content and existing type on the vulcanization process, crosslinking network structure and mechanical properties of vulcanized NR were analyzed, and the following conclusions are drawn:

Both free and bonded proteins can promote NR vulcanization and reduce the vulcanization time; the bonded proteins have a more significant effect on the vulcanization time, although their content is lower than that of the free proteins. Both of them can increase the crosslinking density of vulcanized rubber, and the effect of the free proteins on the crosslinking density is more significant; the removal of the free proteins reduces the crosslinking density from 2.35 × 10^−4^ mol/cm^3^ to 1.52 × 10^−4^ mol/cm^3^, and the reduction in the bonded proteins has less of an effect on the crosslinking density, which is due to the fact that the bonded proteins are involved in the construction of the rubber gum network, leading to it being more difficult to form zinc salt coordinated complexes to promote the formation of crosslinking points. Both the free proteins and bonded proteins can participate in the construction of the crosslinking network of vulcanized NR, but the amount of the bonded proteins is not large enough to achieve the optimum crosslinking density, so the free proteins need to take part in the formation of the crosslinking network to achieve the optimum crosslink density; thus, the tensile properties of the vulcanized NR can be optimized. This conclusion can give guidance on preparing NR with different types of proteins and regulating NR properties by tuning proteins, which will promote the creation of vulcanized NR with excellent mechanical properties.

## Figures and Tables

**Table 1:** Nitrogen content of natural rubber with different protein content.

Sample	Nitrogen Content %	Protein Content %
NR-1	0.537 ± 0.004	3.356 ± 0.025
NR-2	0.157 ± 0.001	0.984 ± 0.006
NR-3	0.099 ± 0.001	0.619 ± 0.006
NR-4	0.032 ± 0.001	0.198 ± 0.006
NR-5	0.022 ± 0.003	0.135 ± 0.017

**Table 2:** Vulcanization characteristic parameters of natural rubber with different protein content.

Samples	M_H_/dN·m	M_L_/dN·m	t_10_/min	t_90_/min
NR-1	7.14	0.72	1.7	12.2
NR-2	6.64	0.71	3.4	10.2
NR-3	6.12	0.55	8.8	18.9
NR-4	6.12	0.62	11.1	22.8
NR-5	6.04	0.63	11.8	24.1

**Table 3:** Mechanical property parameters of natural rubber with different protein content.

Projects	NR-1	NR-2	NR-3	NR-4	NR-5
Tensile strength (MPa)	27.80 ± 0.92	30.81 ± 1.57	27.45 ± 1.06	27.64 ± 2.08	26.70 ± 1.58
100% stress (MPa)	1.11 ± 0.02	0.86 ± 0.03	0.77 ± 0.01	0.70 ± 0.02	0.68 ± 0.03
300% stress (MPa)	2.82 ± 0.05	2.04 ± 0.06	1.77 ± 0.02	1.64 ± 0.06	1.58 ± 0.09
500% stress (MPa)	10.31 ± 0.4	5.03 ± 0.51	4.02 ± 0.07	3.7 ± 0.50	3.28 ± 0.43
Elongation at break (%)	656 ± 7	752 ± 20	738 ± 9	756 ± 26	753 ± 30

**Table 4:** Crosslinking density of natural rubber with different protein content.

Samples	*Q* (%)	$V_{c}\;(10^{-4}$ mol/cm^3^)	*M_C_* (kg/mol)
NR-1	4.06	2.35	3.88
NR-2	4.61	1.74	5.25
NR-3	4.89	1.52	6.02
NR-4	4.95	1.47	6.19
NR-5	4.97	1.46	6.24

**Table 5:** Network structure parameters of each sample.

Samples	NR-1	NR-2	NR-3	NR-4	NR-5
*G_c_* (MPa)	0.52	0.39	0.33	0.32	0.32
*G_e_* (MPa)	0.24	0.20	0.22	0.18	0.14
*V_c_*$\times 10^{-4}$ (mol/mL)	3.12	2.37	1.99	1.90	1.95
*M_c_* (g/mol)	2978	3916	4664	4897	4776
*R_c_* (nm)	4.05	4.64	5.07	5.19	5.13
N	28	37	44	46	45
*d*_0_ (nm)	2.33	2.54	2.44	2.74	3.07
*n_e_*	9	11	10	13	16

## Data Availability

Data are contained within the article.
